# Raloxifene ameliorates cartilage and subchondral bone microstructural degeneration in the ovariectomy-induced spontaneous postmenopausal osteoarthritis

**DOI:** 10.3389/fgene.2026.1795443

**Published:** 2026-03-30

**Authors:** Ya-Ping Xiao, Mu-Wei Dai, Fa-Ming Tian, Li-Tao Shao, Ming-Jian Bei, Liu Zhang, Zhang-Hua Li

**Affiliations:** 1 The Department of Orthopedic Surgery, Wuhan Third Hospital, Tongren Hospital of Wuhan University, Wuhan, China; 2 Department of Orthopedic Surgery, The Affiliated Hospital of North China University of Science and Technology, Tangshan, Hebei, China; 3 Medical Research Center, North China University of Science and Technology, Tangshan, Hebei, China

**Keywords:** cartilage, osteoarthritis, osteoporosis, ovariectomy, raloxifene, subchondral bone

## Abstract

The prevalence and incidence of osteoarthritis (OA) increase significantly in women after menopause, indicating an important role of estrogen in the pathogenesis of OA. This type of OA is termed postmenopausal OA. This study aimed to investigate the feasibility of using bilateral ovariectomy (OVX) in adult SD rats to simulate the human postmenopausal OA model and evaluate the effect of early raloxifene (RAL) intervention on this model. Twenty-four SD rats were randomly divided into 4 groups: Baseline group, Sham + V group, OVX + V group, and OVX + RAL group. Rats in the Baseline group were euthanized for sample collection at the start of the experiment. Rats in the OVX + V and OVX + RAL groups underwent bilateral OVX, while those in the Sham + V group received a sham operation without actual ovarian resection. After surgery, the OVX + RAL group was given RAL (6.2 mg/kg·day) by gavage, and the OVX + V and Sham + V groups received an equal volume of normal saline. Samples were collected 3 months after drug administration. Micro-CT was used to determine the bone histomorphometry of the right proximal tibia. Following Micro-CT analysis, the right knee joints of all animals were decalcified for 8–12 weeks, embedded in paraffin, and sectioned. The sections were subjected to toluidine blue staining and immunohistochemical staining for collagen-II, Caspase-3, and matrix metalloproteinase-13 (MMP-13). The toluidine blue-stained sections were scored using the OARSI histological scoring system, and the positive protein expression in immunohistochemical staining was evaluated using the IOD. The OARSI score revealed that the degree of cartilage degeneration in the OVX + V group was more severe than that in the Sham + V group and the OVX + RAL group. The expression of collagen-II in the OVX + V group was significantly lower than that in the Sham + V group and the OVX + RAL group, while the expressions of MMP-13 and Caspase-3 increased. Micro-CT revealed that the microstructure of subchondral bone in the OVX + V group deteriorated compared with the Sham + V group, while that in the OVX + RAL group improved compared with the OVX + V group. Compared with the Baseline group, the microstructure of subchondral bone and cartilage in the Sham + V group was somewhat degraded. We reached a conclusion that OVX-induced degeneration of subchondral bone and articular cartilage is relatively mild, suggesting that 6-month-old OVX rats are a mild model of postmenopausal OA. RAL can delay OVX-induced postmenopausal subchondral bone and cartilage degeneration. Notably, this study further clarifies the protective effect of RAL on the medial joint capsule and refines the regulatory mechanism of RAL on subchondral bone microstructure in mild postmenopausal OA, which supplements the existing research on RAL in OA intervention.

## 1 Introduction

Osteoarthritis (OA) is a degenerative joint disease characterized primarily by the microstructure degeneration of articular cartilage and subchondral bone, with synovitis and osteophyte formation in the advanced stage (Yang et al., 2024). Eventually, it affects the entire joint organ, leading to joint dysfunction. OA is also one of the leading causes of disability in the elderly and has become the fourth most disabling disease globally (Courties et al., 2024; Xu et al., 2025). After the age of 50, women enter menopause, and the prevalence and incidence of OA increase significantly. OA occurring in postmenopausal women is named postmenopausal OA (Ho et al., 2024; Xu et al., 2025).

Currently, the relationship between estrogen levels and OA has been gradually clarified by researchers. Estrogen alleviates OA progression in ovariectomized mice (Li et al., 2022). In addition, estrogen receptors have been identified in chondrocytes and bone tissue, enabling estrogen to act on articular cartilage and subchondral bone (Steppe et al., 2022). ERα gene polymorphisms may be associated with the risk of developing OA (Chen et al., 2024). These lines of evidence suggest that changes in endogenous estrogen levels may be one of the etiological factors of OA. Estrogen replacement therapy remains controversial due to its side effects. Selective estrogen receptor modulators (SERMs) are a promising alternative that can reduce the clinical risks of estrogen replacement therapy (Xiao et al., 2016).

Raloxifene (RAL) is a SERM that exerts estrogen agonist effects on bone tissue. RAL can reduce bone turnover, inhibit bone loss, promote bone formation, and increase bone mineral density (Shogry et al., 2025). RAL may have dual effects on OA joints: directly acting on subchondral bone and directly/indirectly acting on articular cartilage, synovium, muscles, and other joint tissues to maintain joint health as a whole and exert a protective effect on joint tissues. Existing *in vivo* studies have confirmed the protective effect of RAL on OVX-induced OA in rats (Bei et al., 2020; Ping et al., 2021), but these studies mainly focused on patellofemoral joint OA or osteoporotic OA with severe degeneration, and the regulatory effect of RAL on mild postmenopausal OA, especially the protective effect on joint capsule and medial tibial plateau subchondral bone microstructure, remains unclear. In addition, the correlation between RAL intervention and age-related mild cartilage degeneration in adult rats has not been reported. Therefore, this study used 6-month-old adult rats as experimental subjects, performed bilateral ovariectomy (OVX) to simulate the decrease in estrogen levels in postmenopausal elderly women, and exclude the interference of severe age-related degeneration (12–18 months old rats), and induce mild bone loss and concurrent postmenopausal OA. RAL was then used for early intervention to evaluate the therapeutic effect of RAL on mild postmenopausal OA and its protective effect on joint capsule tissue.

Recent studies have further emphasized the importance of subchondral bone-cartilage mechanical balance in OA progression (Yang et al., 2024) and the potential of anti-osteoporosis drugs in OA intervention (Wei et al., 2024; Sheng et al., 2025). In addition, the organ-joint axis theory has been proposed to explain the interactive damage of joint tissues in OA (Yang et al., 2024), which provides a new theoretical basis for exploring the overall protective effect of RAL on joint tissues. Meanwhile, Wen et al. (2024) found that subchondral bone remodeling disorder is a key initiator of mild OA, which further supports the research significance of this study on mild postmenopausal OA model.

## 2 Methods

### 2.1 Animals

Twenty-four 6-month-old female SPF-grade SD rats (Batch Number: SDXK Jing, 2015-0001, Vital River Experimental Animal Technical Co., Ltd., Beijing, China) were used for the experimental study. The selection of 6-month-old SD rats was based on the following basis: (1) 6-month-old SD rats are in the adult stage with stable physiological status, and their cartilage and subchondral bone have no obvious age-related severe degeneration, unlike 12–18 months old aged rats; (2) It can exclude the interference of severe age-related degeneration on the experimental results, and accurately reflect the effect of estrogen deficiency on joint tissue damage. This experimental protocol was conducted in accordance with the “Guide for the Care and Use of Laboratory Animals” and approved by the Animal Care and Use Committee of North China University of Science and Technology. Before the experiment, all rats were acclimated for 1 week at a temperature of 20 °C ± 1 °C with a 12-hour light/dark cycle and fed with SPF-grade rat growth and reproduction feed. No more than three rats were housed per cage to ensure sufficient activity and avoid biting.

The SD rats were randomly numbered and marked, and then the numbers were randomly grouped by computer using the random number method. A total of 4 groups were divided, with 6 rats in each group, namely: the baseline group (n = 6), the control group (Sham + V group, n = 6), the model group (OVX + V group, n = 6), and the experimental group (OVX + RAL group, n = 6). All SD rats were fed with regular feed without adding any substances that might affect the experiment, except to ensure normal growth and development. Rats in the baseline group were euthanized for sample collection at 6 months of age. Rats in the OVX + V and OVX + RAL groups underwent bilateral OVX, while those in the Sham + V group received a sham operation involving only exposure of the abdominal cavity and ovaries without OVX.

### 2.2 Surgical procedure

On the night before surgery, rats were fasted and deprived of water to prevent aspiration during the operation. The 7% chloral hydrate injection was prepared in advance. Intraperitoneal injection of anesthesia was used at a dose of 5 mL/kg body weight. After anesthesia, the hair in the surgical area and within 3 cm around it was removed using an electric clipper for skin preparation. The operating room was disinfected in advance, and the indoor temperature was maintained at approximately 25 °C. After the rats were successfully anesthetized, they were fixed on the operating table in a lateral position. The surgical area was routinely disinfected with povidone-iodine, and sterile surgical drapes were placed. After the start of the operation, a longitudinal incision of approximately 1.5 cm was made at two transverse fingers below the costal margin on the left and right sides of the rat. The skin, subcutaneous tissue, abdominal muscles, and peritoneum were incised layer by layer to expose the abdominal cavity. The ovaries were located and separated, gently clamped out of the abdominal cavity with gentle manipulation to avoid damaging the ovaries and surrounding adipose tissue and preventing vascular rupture and intra-abdominal hemorrhage. Bilateral ovarian ligation and resection were performed sequentially. After the operation, the wound was irrigated with normal saline, and the wound was sutured layer by layer to the skin. The Sham + V group was subjected to the same surgical procedures as the OVX group, including the same incision location, anesthesia dose, layer-by-layer incision of abdominal tissues, exposure of ovaries, and layer-by-layer suture of the wound; the only difference was that the ovaries were not ligated and resected, to ensure the consistency of surgical stress between the two groups. The wound was disinfected with povidone-iodine daily to prevent infection. Due to the paralytic ileus effect of chloral hydrate, the rats were fasted for 1 day after surgery to prevent paralytic ileus, and then allowed to eat and move freely thereafter.

### 2.3 Drug administration

One day after surgery, rats in the OVX + RAL group were given RAL hydrochloride tablets (6.2 mg/kg·day, Jiangsu Hengrui Medicine Co., Ltd., Lianyungang, Suzhou, China) by gavage. The dose of 6.2 mg/kg·day was determined based on the reference of Ping et al. (Ping et al., 2021), which was the optimal effective dose for RAL to intervene in OVX-induced osteoporotic OA in rats in previous studies, and this dose was also verified to have no obvious toxic and side effects on rat liver and kidney functions. The OVX + V group and Sham + V group were given an equal dose of normal saline by gavage (Ping et al., 2021). All rats were euthanized for sample collection 3 months after the OVX. The selection of 3-month drug intervention period was based on pre-experimental results (Bei et al., 2020; Ping et al., 2021; Wei et al., 2024): (1) Pre-experiments showed that OVX-induced estrogen deficiency in rats could cause stable mild degeneration of cartilage and subchondral bone within 3 months, which was consistent with the characteristics of early postmenopausal OA in humans; (2) The protective effect of RAL on joint tissue could be stably observed within this period, and no severe degeneration would mask the drug effect; (3) This period is a commonly used effective observation period for OA intervention studies in rats, which is conducive to the comparison with existing studies.

### 2.4 Micro-CT detection of subchondral bone structure

Samples were recorded using a digital camera (Canon 550D; Canon, Tokyo, Japan) and then directly fixed in 100% ethanol for Micro-CT detection. Micro-CT (SkyScan1176, Kontich, Belgium) was used to scan and collect data on the subchondral bone specimens of the proximal tibia of the knee joint at 40 kV and 25 W. The scanned images were analyzed and three-dimensionally reconstructed using Micro-CT supporting software including NRecon, CT Analyser, CT vox, and DataViewer (Kontich, Belgium). The region of interest (ROI) was selected as 0.5 mm under the medial plateau cartilage based on the following rational basis: (1) The medial tibial plateau is the main weight-bearing part of the rat knee joint, and it is the most susceptible to degeneration after estrogen deficiency, which is consistent with the pathological characteristics of human postmenopausal OA; (2) 0.5 mm under the cartilage is the key area of subchondral bone that directly participates in the mechanical transmission with cartilage, and its microstructure changes can directly reflect the interaction between subchondral bone and cartilage; (3) This ROI selection method is a commonly used standard method in rat knee subchondral bone Micro-CT research, which ensures the repeatability and comparability of the experiment. The ROI was selected as 0.5 mm under the medial plateau cartilage, with a resolution of 9 μm and a volume of 2 mm × 2 mm × 0.4 mm cube. In this experiment, subchondral bone microstructure indices including bone volume fraction (BV/TV), trabecular number (Tb.N), trabecular separation (Tb.Sp), intersection surface (I.S), structural model index (SMI), trabecular thickness (Tb.Th), degree of anisotropy (DA), bone surface to bone volume ratio (BS/BV) and bone surface density (BS/TV) were measured to analyze the changes in subchondral bone microstructure.

### 2.5 Macroscopic analysis

Rat knee joint specimens were fixed in 4% neutral buffered formalin for at least 1 week, decalcified with 15% EDTA. The decalcifying solution was replaced weekly, with a general decalcification time of 8–12 weeks. All specimens were embedded in paraffin and sectioned at a thickness of 4 μm. The sections were stained with toluidine blue, and the morphological changes of the knee joint were observed under a light microscope. Histopathological scoring was performed according to the latest knee joint histopathological scoring guidelines (OARSI score) issued by the Osteoarthritis Research Society International (OARSI) (Gerwin et al., 2010). Since the medial tibial plateau is the main weight-bearing part of the knee joint and the most severely worn part in OA, the medial tibial plateau was selected for OARSI scoring in this experiment. The OARSI scoring and analysis of all samples were performed by two professional pathologists who independently scored the toluidine blue staining results. The main observation indicators included cartilage matrix loss width, total cartilage degeneration width, significant cartilage degeneration width, zonal depth ratio of lesions, osteophyte score, calcified cartilage and subchondral bone damage score, and medial joint capsule repair.

### 2.6 Immunohistochemistry

Immunohistochemical staining for collagen-II, Caspase-3, and matrix metalloproteinase-13 (MMP-13) in cartilage was performed. Semi-quantitative analysis of immunohistochemically stained specimens was performed using Image-Pro Plus 6.0 software, converting the intensity of immunohistochemical staining into integrated optical density (IOD), and the operation process was similar to the immunoreactive score (IRS). Semi-quantitative analysis of IOD values was performed on all immunohistochemically stained specimens in this experiment (Bei et al., 2020). Two professional pathologists independently measured the results of immunohistochemical staining, and the IOD value represents the intensity of immunohistochemical staining. The successful induction of estrogen deficiency in OVX rats was verified by the following indirect indicators: (1) Micro-CT results showed that OVX + V group had significant subchondral bone loss or decreased BV/TV and Tb.N, compared with Sham + V group, which is a typical characteristic of estrogen deficiency-induced osteoporosis; (2) Histological results showed that OVX + V group had obvious cartilage degeneration compared with Sham + V group, which is consistent with the joint damage caused by estrogen deficiency; (3) The above indicators were reversed after RAL intervention, which further confirmed the successful establishment of estrogen deficiency model.

### 2.7 Statistical analysis

SPSS 19.0 software was used for data processing. After testing the normality of the data in each group using the Kolmogorov-Smirnov test and the homogeneity of variance using the Student-newman-keuls test, the data in each group that conformed to normality and homogeneity of variance were analyzed using one-way analysis of variance (ANOVA), and the LSD-t was used for pairwise comparison between groups (collagen-II immunohistochemical staining, total cartilage degeneration width, zonal depth ratio of lesions, osteophyte score, medial joint capsule repair, BV/TV, Tb.Sp, BS/BV, and SMI). Data in each group that did not conform to normality or homogeneity of variance were analyzed using the Kruskal–Wallis H test, followed by the Mann-Whitney U test for pairwise comparisons (MMP-13 and Caspase-3 immunohistochemical staining, significant cartilage degeneration width, cartilage matrix loss width, calcified cartilage and subchondral bone damage score, Tb.Th, Tb.N, BS/TV, I.S, and DA). All pairwise comparisons were based on the results of multi-group comparison first, and the asterisk (*) in the figures only indicates the statistical significance of the pairwise comparison between the OVX + V group (*P* < 0.05), which is a supplementary description of the multi-group ANOVA results, not the result of simple Student’s t-test. All experimental data were expressed as mean ± standard deviation (X ®±SD), with α = 0.05 as the significance test level, and *P* < 0.05 indicated a statistically difference. All graphs in this study have been supplemented with individual data points to reflect the data distribution characteristics of each group.

## 3 Results

All SD rats successfully completed the modeling surgery and the experimental process, and a total of 24 SD rats were included in this study.

### 3.1 Macroscopic analysis results

**FIGURE 1:**
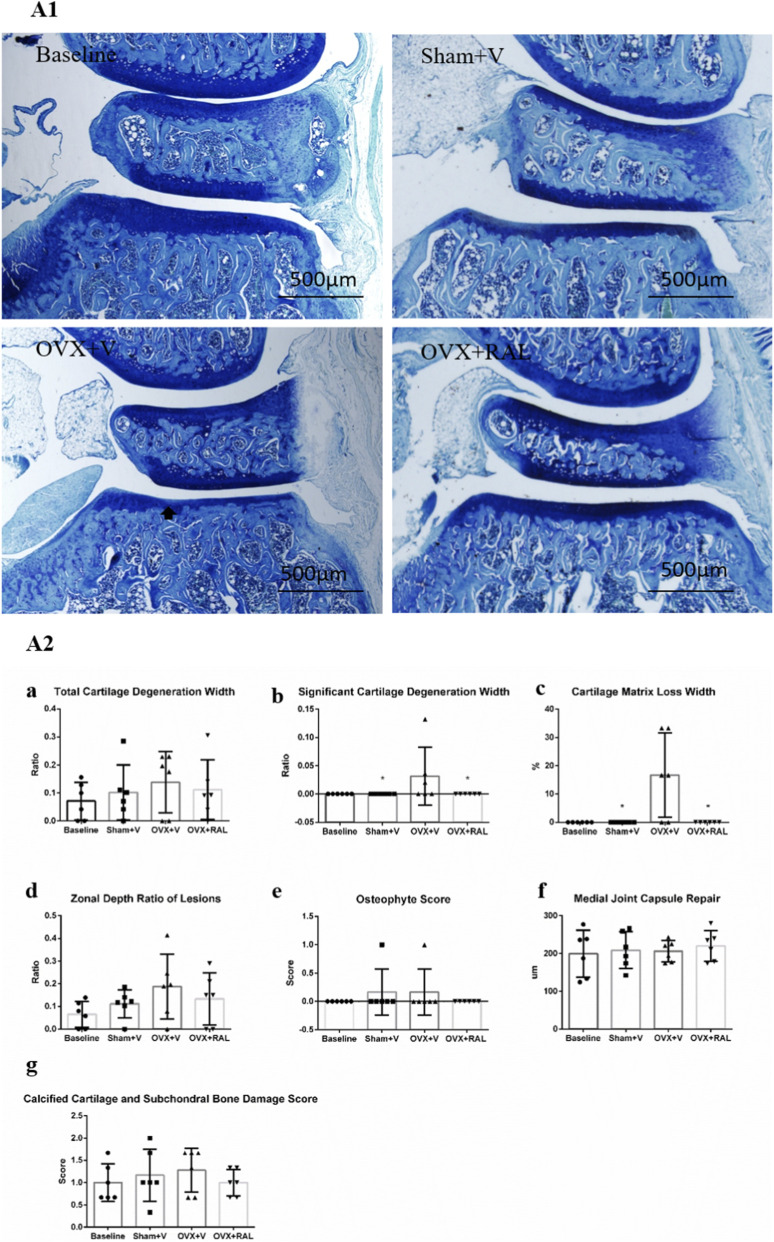
Toluidine blue staining of articular cartilage (40×) and OARSI scoring of toluidine blue staining between groups ( $\bar{X}$ ±SD): **(A1)** Toluidine blue staining of articular cartilage: The degeneration of articular cartilage in the OVX + V group was significantly more severe than that in the baseline group, the Sham + V group, and the OVX + RAL group; **(A2a)** The OARSI scoring of total cartilage degeneration width; **(A2b)** The OARSI scoring of significant cartilage degeneration width; **(A2c)** The OARSI scoring of cartilage matrix loss width; **(A2d)** The OARSI scoring of zonal depth ratio of lesions; **(A2e)** The OARSI scoring of osteophyte score; **(A2f)** The OARSI scoring of medial joint capsule repair; **(A2g)** The OARSI scoring of calcified cartilage and subchondral bone damage score. Black arrow: Local degeneration of articular cartilage; * Compared with the OVX + V group, *P* < 0.05.

Light microscopic observation of sections showed that compared with the baseline group, Sham + V group, and OVX + RAL group, the OVX + V group had mild degenerative changes in the cartilage surface, shallow staining in the superficial layer of the weight-bearing area, partial loss of cartilage matrix and chondrocytes, slightly abnormal chondrocyte morphology, slightly blurred articular cartilage layers, occasional osteophyte formation at the edges, slightly disordered tidemark continuity, increased basophilic cells at the tidemark, mild thickening of subchondral bone, and changes in bone marrow mesenchymal cells (Figure 1A1).

The cartilage matrix loss width and significant cartilage degeneration width in the OVX + V group were significantly higher than those in the Sham + V group and OVX + RAL group (*P* < 0.05), while there was no significant difference between the Sham + V group and the baseline group (*P* > 0.05, Figures 1A2c,b). The total cartilage degeneration width, zonal depth ratio of lesions and calcified cartilage and subchondral bone damage score in the OVX + V group were slightly increased compared with the Sham + V group and OVX + RAL group (*P* > 0.05, Figures 1A2a,d,g), while there was slightly increased in the Sham + V group compared with the baseline group (*P* > 0.05). OARSI scoring of osteophytes showed that only 1 sample respectively in the Sham + V group and OVX + V group had mild osteophytes with a score of 1, and the osteophyte score of other samples was 0; no osteophytes were found in samples from the OVX + RAL group and baseline group, and the osteophyte score of all samples was 0. There was no significant difference between groups (*P* > 0.05, Figure 1A2e). Compared with the OVX + V group, the OVX + RAL group and Sham + V group had a slightly increased medial joint capsule repair (*P* > 0.05, Figure 1A2f), and there was no significant difference between the Sham + V group and the baseline group (*P* > 0.05).

### 3.2 Immunohistochemical staining results

**FIGURE 2:**
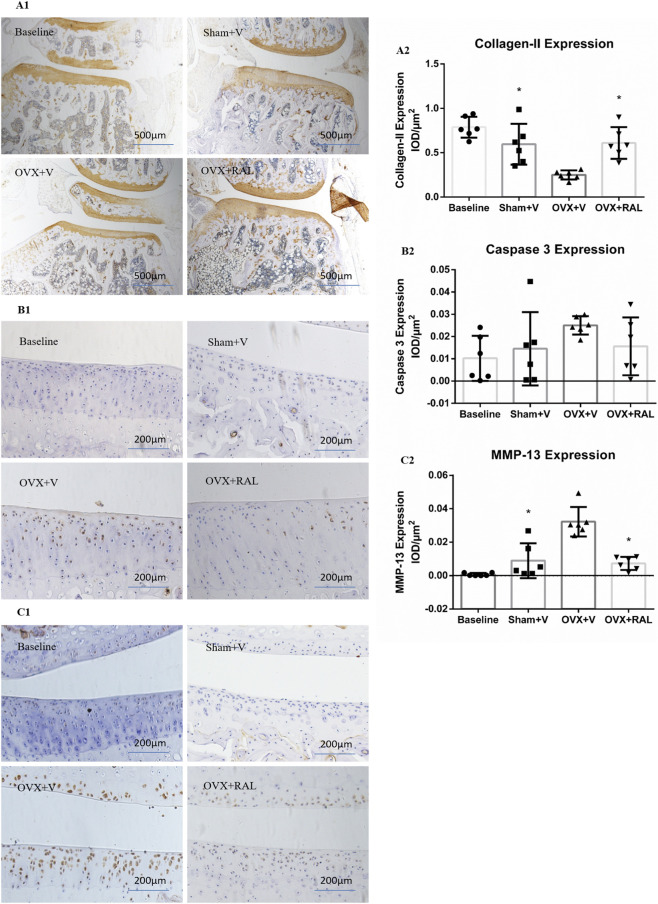
The immunohistochemisty staining of collagen-II, caspase-3 and MMP-13 of articular cartilage (40×) and the results of IOD analysis of immunohistochemical staining ( $\bar{X}$ ±SD): **(A1)** The immunohistochemisty staining of collagen-II; **(A2)** The results of IOD analysis of immunohistochemisty staining of collagen-II; **(B1)** The immunohistochemisty staining of caspase-3; **(B2)** The results of IOD analysis of immunohistochemisty staining of caspase-3; **(C1)** The immunohistochemisty staining of MMP-13; **(C2)** The results of IOD analysis of immunohistochemisty staining of MMP-13. * Compared with the OVX + V group, *P* < 0.05; & vs. Sham + V group, *P* < 0.05.

Compared with the Sham + V group and OVX + RAL group, the positive expression of collagen-II immunohistochemical staining in the OVX + V group was significantly decreased (*P* < 0.05, Figures 2A1,A2). Compared with the baseline group, the positive expression of collagen-II immunohistochemical staining in the Sham V group was slightly decreased (*P* > 0.05). Compared with the Sham + V group and OVX + RAL group, the positive expression of Caspase-3 immunohistochemical staining in the OVX + V group was slightly increased (*P* > 0.05, Figures 2B1,B2). There was slightly increased in expression level between the Sham + V group compared with the baseline group (*P* > 0.05). Compared with the Sham + V group and OVX + RAL group, the positive expression of MMP-13 immunohistochemical staining in the OVX + V group was significantly increased (*P* < 0.05, Figures 2C1,C2). Compared with the baseline group, the positive expression in the Sham + V group was slightly increased (*P* > 0.05).

The reason for the different expression levels of MMP-13 and Caspase-3 is that MMP-13 is a key enzyme directly involved in cartilage matrix degradation (type II collagen hydrolysis), and estrogen deficiency can directly upregulate its expression through the ERα pathway, leading to significant increase; while Caspase-3 is a key factor of chondrocyte apoptosis, and estrogen deficiency only induces mild chondrocyte apoptosis in the mild OA model of this study, so its expression is only slightly elevated without statistical significance.

### 3.3 Micro-CT measurement results

**FIGURE 3:**
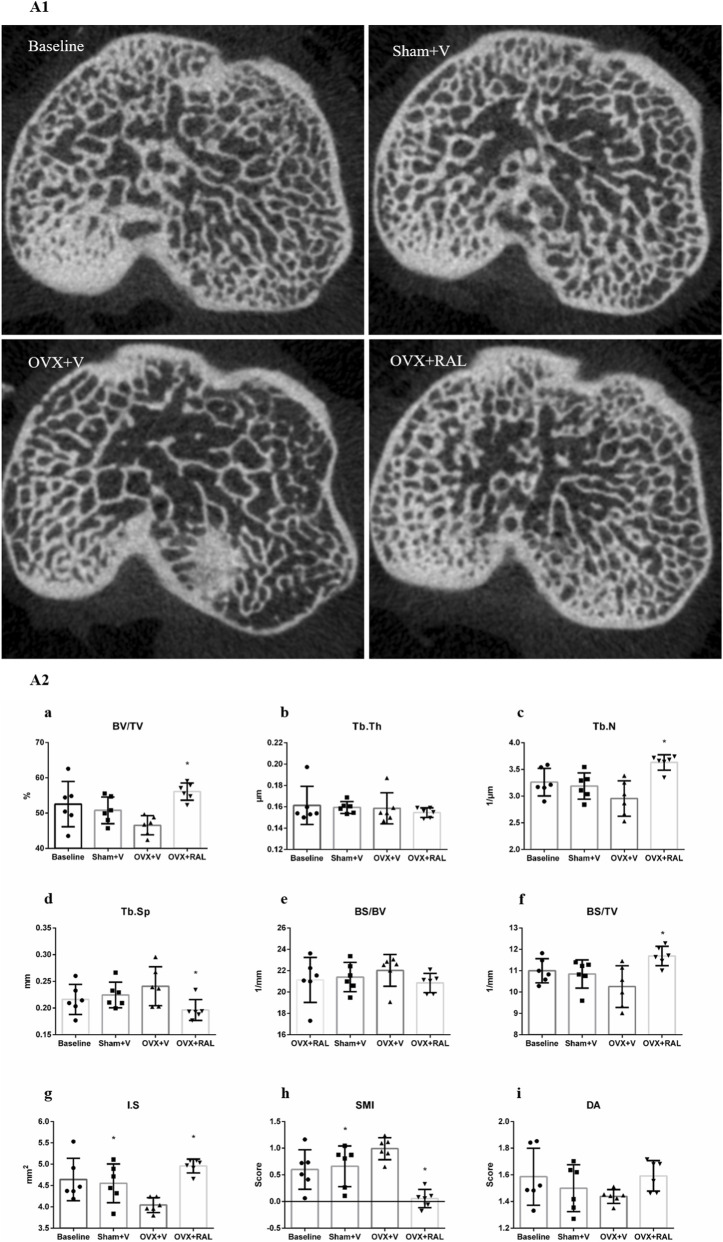
The scanning sections of Micro-CT in subchondral bone and the statistical results of Micro-CT sections ( $\bar{X}$ ±SEM): **(A1)** The scanning sections of Micro-CT in subchondral bone between groups; **(A2a)** Bone volume fraction (BV/TV); **(A2b)** Trabecular thickness (Tb.Th); **(A2c)** Trabecular number (Tb.N); **(A2d)** Trabecular separation (Tb.Sp); **(A2e)** Bone surface to bone volume ratio (BS/BV); **(A2f)** Bone surface density (BS/TV); **(A2g)** Intersection surface (I.S), **(A2h)** Structural model index (SMI); **(A2i)** Degree of anisotropy (DA). * Compared with the OVX + V group, *P* < 0.05.

Gross observation showed that compared with the Sham + V group and OVX + RAL group, the OVX + V group had a significant decrease in trabecular number, sparse trabeculae, thinning of trabeculae, and reduced interconnection of trabeculae (Figure 3A1). Compared with the baseline group, the Sham + V group had a slight decrease in trabecular number and the interconnection of trabeculae.

The values of BV/TV, Tb.N, and BS/TV in the OVX + V group were significantly lower than those in the OVX + RAL group (*P* < 0.05, Figures 3A2a,c,f), and lower than those in the Sham + V group (*P* > 0.05). The values of BV/TV, Tb.N, and BS/TV in the Sham + V group were slightly lower than those in the baseline group (*P* > 0.05). The I.S in the OVX + V group was significantly lower than that in the Sham + V group and OVX + RAL group (*P* < 0.05, Figure 3A2g). The value of I.S in the Sham + V group showed a decreasing trend compared with the baseline group (*P* > 0.05).

The Tb.Sp and SMI in the OVX + V group were significantly higher than those in the OVX + RAL group (*P* < 0.05, Figures 3A2d,h), and these values in the OVX + V group showed an increasing trend compared with the Sham + V group (Tb.Sp *P* > 0.05, SMI *P* < 0.05). These values of Tb.Sp and SMI in the Sham + V group showed an increasing trend compared with the Baseline group (*P* > 0.05). The BS/BV in the OVX + V group were slightly higher than those in the OVX + RAL and Sham + V group (*P* > 0.05, Figure 3A2e), and in the Sham + V group showed an increasing trend compared with the baseline group (*P* > 0.05). The DA in the OVX + V group were slightly lower than those in the OVX + RAL and Sham + V group (*P* > 0.05, Figure 3A2i), and in the Sham + V group showed an decreasing trend compared with the baseline group (*P* > 0.05). There were no statistically differences in Tb.Th between the OVX + V group, Sham + V group, Baseline group, and OVX + RAL group (*P* > 0.05, Figure 3A2b).

## 4 Discussion

This study found that compared with the Sham + V group, the OVX + V group had increased morphological cartilage damage, decreased collagen-II expression indicating cartilage matrix loss, increased Caspase-3 expression indicating increased chondrocyte apoptosis, and increased MMP-13 expression indicating increased cartilage-degrading enzymes. These results suggest that OVX can induce degeneration of cartilage and cartilage matrix. Furthermore, Micro-CT results showed that compared with the Sham + V group, the OVX + V group had decreased BV/TV, Tb.N, BS/TV, I.S and DA, and increased SMI, BS/BV and Tb.Sp, indicating reduced subchondral bone trabeculae, disordered structure, and increased severity of subchondral bone osteoporosis. The degeneration of cartilage and subchondral bone confirms that bilateral OVX can induce spontaneous postmenopausal OA in adult rats, successfully establishing an adult rat model of postmenopausal OA. This study found that by comparing with the OVX + V group, RAL can reduce articular cartilage damage, decrease the expression of MMP-13 and Caspase-3 and increase the expression of collagen-II. Therefore, RAL can alleviate the degeneration of cartilage and cartilage matrix in postmenopausal OA rats to a certain extent. Compared with the OVX + V group, the OVX + RAL group had increased BV/TV, Tb.N, BS/TV, I.S and DA, and decreased SMI, BS/BV and Tb.Sp, indicating that RAL can alleviate subchondral bone osteoporosis and structural degeneration. In addition, OARSI scoring found that RAL improved the abnormal changes in the medial joint capsule repair induced by OVX, suggesting that RAL may also have a protective effect on the joint capsule. The joint is an organic whole, and joint tissues interact with each other. RAL acts on all joint tissues and exerts a protective effect on OA joints as a whole. The Sham + V group showed an increasing trend in cartilage and subchondral bone degeneration compared with the baseline group, suggesting that cartilage and subchondral bone degeneration gradually worsens with age in adult rats.

In the occurrence and development of OA, subchondral bone abnormalities, especially increased bone turnover and destruction of bone microstructure, disrupt the mechanical balance between subchondral bone and cartilage, playing an important role in articular cartilage damage (Bellido et al., 2010; Bellido et al., 2011). Conversely, improvements in subchondral bone turnover and structure can delay the progression of OA (Bellido et al., 2010; Bellido et al., 2011). OA may have a new phenotype, namely osteoporotic OA, which is characterized by high subchondral bone turnover in the early stage, accompanied by subchondral bone loss (Sheng et al., 2025). Anti-osteoporosis drugs regulate bone turnover, increase bone mineral density, act on subchondral bone, improve subchondral bone turnover and microstructure, regulate the mechanical balance between subchondral bone and cartilage, and may have a positive effect on articular cartilage or OA (Xiao et al., 2016; Ho et al., 2024; Wei et al., 2024). The advanced stage of OA is dominated by subchondral bone sclerosis, with local cyst formation and osteophyte formation (Xiao et al., 2016; Wei et al., 2024; Sheng et al., 2025).

Calvo et al. (2007) reported that estrogen deficiency-induced osteoporosis alters subchondral bone structure and increases the severity of cartilage damage. Mutations in aromatase gene involved in estrogen secretion and estrogen receptor gene are associated with the severity of OA in large lower limb joints. ERα gene polymorphisms may be associated with the risk of developing OA (Ren et al., 2015). These lines of evidence suggest that changes in endogenous estrogen levels may be one of the etiological factors of OA. Therefore, this experiment selected 6-month-old adult SD rats and performed bilateral OVX to simulate the decrease in estrogen levels after menopause. Compared with young rats (3-month-old or 2-month-old rats), this eliminates the impact of age factors on the model, making the experimental model more in line with physiological changes, better simulating the postmenopausal estrogen deficiency state in humans, and laying the foundation for subsequent experiments. Compared with 12–18 months old aged rats, 6-month-old rats have the following limitations: (1) The OA model induced by OVX is only mild degeneration, which cannot simulate the severe postmenopausal OA in elderly women (over 70 years old); (2) The age-related degeneration of joint tissue is not obvious, so the synergistic effect of estrogen deficiency and age on OA cannot be studied; (3) The protective effect of RAL observed in this study is only for mild OA, and its effect on severe OA needs to be further verified. This study found that bilateral OVX in rats successfully induced the development of postmenopausal OA, further confirming the rationality and feasibility of this experimental animal model (Høegh-Andersen et al., 2004).

Improving the bone mass and microstructure of subchondral bone, improving the mechanical transmission between subchondral bone and cartilage, and alleviating abnormal stress loading can reduce abnormal changes both cartilage and subchondral bone, and ultimately may have a potential beneficial effect on OA (Ho et al., 2024). In a clinical trial, RAL not only reduced the level of cartilage metabolic marker CTX-II in the serum of postmenopausal women but also decreased the level of bone metabolic marker CTX-I, indicating that RAL has dual effects on cartilage and subchondral bone (Karsdal et al., 2012). In in vitro experiments, RAL significantly reduced the expression of OA-related genes and Caspase-3, decreased the protein expression of NO, MMP-13, and MMP-3, increased the expression of Aggrecan and type II collagen genes, and increased the deposition of collagen and sulfated glycosaminoglycans in cartilage, thereby enhancing the mechanical properties of cartilage (Kavas et al., 2013). Similarly, RAL decreased MMP-13, ADAMTS-4, and caspase-3 and increased type II collagen, aggrecan protein, and mRNA levels (Bei et al., 2020). These findings indicate that RAL treatment can delay the progression of PFJOA disease (Bei et al., 2020). A study shown that clinical doses of RAL delay the development of osteoporotic OA by inhibiting the overexpression of TGF-β1 in cartilage and regulating subchondral bone metabolism (Ping et al., 2021). However, the clinical implication of this study should be tempered: RAL exerts a protective effect on mild postmenopausal OA in this study, but its effect on clinical severe postmenopausal OA is still unclear; in addition, the drug dose and intervention period in animal experiments cannot be directly translated into clinical practice, and clinical trials with large samples and long-term follow-up are needed to verify. These results suggest that the clinical indications of RAL should be expanded to include the prevention and treatment of early/mild postmenopausal OA.

Age-related cartilage changes are an important factor in the progression of OA, and cartilage degeneration worsens with age (Zhou et al., 2023; Courties et al., 2024). For example, age-related changes in chondrocyte extracellular matrix proteins mainly include non-enzymatic type II collagen cross-linking and shortened aggrecan molecules, leading to increased cartilage stiffness, increased brittleness, and susceptibility to fatigue fractures (Bank et al., 1998; Zhang et al., 2023). These findings suggest that age-related matrix changes play a role in the development of OA. Therefore, with increasing age, cartilage matrix and subchondral bone microstructure may degenerate, leading to increased susceptibility of cartilage and subchondral bone to damage. At the same time, as an organic whole, joint tissues gradually decline in function and structure with age, eventually progressing to OA changes as a whole. Wen et al. (Wen et al., 2024) found that the activation of subchondral bone osteoclasts and the disorder of bone remodeling is the initial factor of mild cartilage degeneration, and this finding is consistent with the results of this study that OVX induces mild subchondral bone loss followed by cartilage degeneration. In addition, the latest study by (Zhao et al., 2025) emphasized the importance of early intervention in mild OA, which further supports the clinical value of RAL in the early prevention of postmenopausal OA.

## 5 Conclusion

OVX-induced degeneration of subchondral bone and articular cartilage is relatively mild, suggesting that 6-month-old OVX rats are a model of postmenopausal OA with mild degeneration. RAL can delay OVX-induced postmenopausal subchondral bone and cartilage degeneration, and may also have a protective effect on the medial joint capsule. Prospective research avenues are as follows: (1) Further explore the protective effect of RAL on severe postmenopausal OA using 12–18 months old aged rat models, and study the synergistic effect of estrogen deficiency and age on OA; (2) Investigate the long-term effect of RAL on postmenopausal OA (6 months or more intervention period) and its potential toxic and side effects on joint tissues; (3) Explore the molecular mechanism of RAL protecting the medial joint capsule in postmenopausal OA, and clarify the role of ERβ pathway in this process; (4) Conduct clinical trials to verify the effect of RAL on early postmenopausal OA patients, and explore the optimal clinical dose and intervention time; (5) Combine RAL with other anti-OA drugs to study the synergistic therapeutic effect on postmenopausal OA. It should be noted that RAL only shows a protective effect on early/mild postmenopausal OA in this study, and its clinical application needs to be combined with the severity of OA in patients and comprehensive evaluation of estrogen receptor expression.

## Data Availability

The original contributions presented in the study are included in the article/supplementary material, further inquiries can be directed to the corresponding authors.
